# Associations between prehospital defibrillation and outcomes of out-of-hospital cardiac arrests presumed to be caused by hypothermia: A nationwide observational study with epidemiological analysis

**DOI:** 10.1097/MD.0000000000033618

**Published:** 2023-04-28

**Authors:** Tomoyuki Ushimoto, Kenshi Murasaka, Yukihiro Wato, Hideo Inaba

**Affiliations:** a Department of Emergency Medicine, Kanazawa Medical University, Uchinada, Japan; b Kanazawa University, Kanazawa, Japan; c Department of Emergency Medical Sciences, Niigata University of Health and Welfare, Niigata, Japan.

**Keywords:** hypothermia, neurologically favorable 1-month survival, outcomes, out-of-hospital defibrillation, pre-hospital cardiac arrest

## Abstract

This study aimed to clarify the epidemiology of out of-hospital cardiac arrest (OHCA) cases caused by hypothermia. The associations between the presence/absence of shockable initial electrocardiography rhythm, prehospital defibrillation and the outcomes of OHCA were also investigated. This study involved the retrospective analysis of prospectively collected, nationwide, population-based data for OHCA cases caused by hypothermia. One thousand five hundred seventy-five emergency medical service (EMS)-confirmed OHCA cases with hypothermia, recorded between 2013 and 2019, were extracted from the Japanese nationwide database. The primary outcome was neurologically favorable 1-month survival, defined as cerebral performance category 1 or 2. The secondary outcome was 1-month survival. OHCA cases with hypothermia occurred more frequently in the winter. In approximately half (837) of the hypothermic OHCA cases, EMS was activated in the morning (6:00 am to 11:59 am). Shockable initial electrocardiogram rhythms were recorded in 30.8% (483/1570) of cases. prehospital defibrillation was attempted in 96.1% (464/483) of cases with shockable rhythms and 25.8% (280/1087) of cases with non-shockable initial rhythms. EMS-witnessed cases, prolonged transportation time intervals and prehospital epinephrine administration were associated with rhythm conversion in cases with non-shockable initial rhythms. Binominal logit test followed by multivariable logistic regression revealed that shockable initial rhythms were associated with better outcomes. prehospital defibrillation was not significantly associated with better outcomes, regardless of the type of initial rhythm (shockable or non-shockable). Transportation to high-level emergency hospitals was associated with better outcomes (adjusted odds ratio: 2.94, 95% confidence interval: 1.66–5.21). In hypothermic OHCA, shockable initial rhythm but not prehospital defibrillation is likely to be associated with better neurologically favorable outcomes. In addition, transport to a high-level acute care hospital may be appropriately considered despite prolonged transport. Further investigation, including core temperature data in analyses, is necessary to determine the benefit of prehospital defibrillation in hypothermic OHCA.

## 1. Introduction

Hypothermia, defined as an involuntary drop in the core body temperature to <35°C, can lead to cardiac arrest and death.^[1]^ Hypothermia can occur in warmer climates and seasons given that it can be caused by a loss of physiological and/or behavioral response to cold exposure. Impaired heat production, increased heat loss and thermoregulatory disorders impair the physiological response to cold exposure, while alcohol, drugs and dementia impair the behavioral response to cold exposure. The epidemiology of out of-hospital cardiac arrest (OHCA) owing to hypothermia may vary based on country or region.^[2]^

The recommended basic and advanced life support treatments for hypothermic OHCA cases differ from those for general OHCA cases. In hypothermic patients, pulse and respiratory rates are slow, breathing is shallow and peripheral vasoconstriction hinders the confirmation of a pulse. Therefore, rescuers providing basic life support assess breathing followed by pulse for a period of 30 to 45 seconds to confirm respiratory arrest, pulseless cardiac arrest or bradycardia profound enough to require cardiopulmonary resuscitation (CPR).^[3,4]^ In addition, rigorous electrocardiogram (ECG) monitoring and whole-body observation are required. Neurologically favorable outcomes after prolonged resuscitation have been reported.^[5]^

Recent guidelines recommend a limited number of sequential defibrillations during the resuscitation of severely hypothermic OHCA patients^[3,4]^; however, the temperature at which defibrillation should first be attempted, and how often it should be attempted, varies between different guidelines.

Defibrillation after rhythm conversion is known to be associated with better outcomes in general OHCA cases with non-shockable initial rhythms, particularly in those with medical causes.^[6]^ In hypothermic patients, sinus bradycardia is reported to be accompanied by atrial fibrillation, followed by ventricular fibrillation and finally asystole as the core temperature decreases.^[7,8]^ In hypothermic cardiac arrest, a non-shockable initial ECG rhythm can be converted to a shockable rhythm in response to rewarming efforts during CPR. However, it has not been fully clarified how frequently non-shockable initial ECG rhythms can be converted to shockable rhythms requiring defibrillation or whether prehospital defibrillation is associated with better outcomes for OHCA cases with hypothermia.

Hypothermia was added as a presumed cause of OHCA to Japan’s nationwide OHCA registry in 2012. Using this prospectively collected nationwide database, we aimed to discover the epidemiological characteristics of OHCA presumed to be caused by hypothermia and to investigate the associations between shockable initial ECG rhythm, prehospital defibrillation and their outcomes.

## 2. Methods

### 2.1. Population and setting

Japan is composed of more than 7000 islands, including 4 major islands: Hokkaido, Honshu, Shikoku, and Kyushu, with a forest coverage of 68.55%. Japan has 4 seasons, and in some regions receive snow in winter. Okinawa and Amami are located in the subtropical zone, with mild temperatures observed even in winter.^[9]^ In 2015, the population of Japan was 127 million, of which 26.6% was aged 65 or over.^[10]^

A total of 6184 ambulances operate across 750 fire departments across Japan. The termination of resuscitation rule is not applied in prehospital settings; unless an OHCA patient is obviously dead, emergency medical service (EMS) personnel continue resuscitation until arrival at the hospital. Japanese EMS personnel manage OHCA cases according to protocols created by regional medical control councils by referring to the Japan Resuscitation Council guidelines.^[11]^ Japanese EMS personnel perform passive external rewarming during the transportation of patients with hypothermia, which involves removing wet clothing and covering the patient with a blanket or insulation. The temperature inside the ambulance is maintained at around 28°C. Japanese EMS personnel can measure axillary temperatures up to 32°C. Although temperature measurement is important in the diagnosis and management of hypothermia, Japanese EMS personnel are not allowed to measure core temperature. Paramedics are allowed to use airway adjuncts and to start a peripheral venous infusion of Ringer’s lactate. However, only authorized and specially trained paramedics are permitted to insert tracheal tubes and administer intravenous epinephrine, and these paramedics are not allowed to administer drugs other than epinephrine. Since 2014, paramedics have also been allowed to perform fluid resuscitation in patients with shock and for those with suspected crush syndrome. In patients with hypothermia, resuscitation procedures are usually performed according to the online physician’s directions.

### 2.2. Data selection

The Japan fire and disaster management agency (FDMA) provides emergency resuscitation statistics data in its possession upon request for their appropriate and effective use. Consent to analyze the OHCA data prospectively collected from January 1, 2013 to December 31, 2019, was obtained from FDMA.

The FDMA database includes Utstein-style information,^[12]^ including information on patient sex, age, witness status, initial ECG rhythm, prehospital defibrillation, prehospital involvement of physician, epinephrine administration, advance airway management, recorded time of witness, bystander CPR initiation, emergency call, EMS vehicle arrival at scenes and hospitals, EMS contact with patient, EMS CPR initiation, epinephrine administration and survival at 1-month with cerebral performance category (CPC).^[13,14]^ Physicians clinically judged whether the OHCA was presumed to be caused by hypothermia in collaboration with the EMS personnel. Fire departments obtained information on the 1-month survival and CPC from hospitals.

From the nationwide database of 879,057 OHCA cases recorded during 2013 to 2019, we extracted 1575 cases of OHCA caused by hypothermia. These data were subjected to epidemiological analysis.

In this study, the initial ECG rhythm was judged to be shockable when a bystander, including non-EMS rescue team members, had performed defibrillation before EMS arrival via public access defibrillation.

In addition, another FDMA transportation database, comprising detailed location, EMS transportation and management, has been available for all emergency transport since 2015, and we combine and reconcile with Utstein-style FDMA data to create a comprehensive database. The results of additional analyses were shown as supplemental materials.

### 2.3. Outcome measures

The primary endpoint was neurologically favorable 1-month survival, defined as CPC 1 or 2. The secondary endpoint was 1-month survival.

### 2.4. Statistical analysis

Differences across groups for nominal variables were assessed using the chi-square test and those for continuous variables were assessed using the Kruskal–Wallis test. The unadjusted or adjusted odds ratios (ORs) and 95% confidence intervals (CIs) were calculated. For each analysis, the null hypothesis was evaluated at a 2-sided significance level of *P* < .05. Adjusted ORs were obtained by simple binominal logit test and multivariable logistic regression analysis. Simple binominal logit test included only 2 factors: initial ECG rhythm (shockable/non-shockable) and prehospital defibrillation. Multivariable logistic regression analysis included the following factors that are well-known to be associated with survival^[15]^: patient sex, age, witnessed status (unwitnessed, bystander-witnessed and EMS-witnessed), initial ECG rhythm (shockable/non-shockable), prehospital defibrillation and time intervals for call receipt-to-arrival at patient (EMS response time interval) and arrival at patient to arrival at the hospital (EMS transportation time interval).

### 2.5. Ethical review

This study was approved by the review board of the Ishikawa Medical Control Council (reference no, 2012-05-2) and conducted in accordance with the Declaration of Helsinki and the Ethical Guidelines issued by the Japanese Ministry of Health, Labour, and Welfare. The requirement for written informed consent was waived because the data analyzed in this study were anonymized and secondary.

#### 1.2.5. Patient and public involvement

Patients or the public were not involved in the design, or conduct, or reporting, or dissemination plans of our study.

## 3. Results

### 3.1. Epidemiology of hypothermic out of-hospital cardiac arrest

The proportion (%) of hypothermic OHCA cases among all OHCA cases recorded in the FDMA database from 2013 to 2019 was highest (0.21%) in 2018, when the lowest temperature was recorded and the amount of snowfall and number of snow days were high.^[16]^ The proportion and incidence (per 100,000 population) of hypothermic OHCA cases varied widely among prefectures (from 0% in Okinawa to 0.62% in Shimane and from 0/100,000 in Okinawa to 5.5/100,000 in Shimane). Approximately 70% (1111/1575) of hypothermic OHCA cases occurred in the winter. In approximately half (837) of hypothermic OHCA cases, EMS was activated by emergency call in the morning (6:00 am to 11:59 am) (Table 1).

**Table 1:** Epidemiology of out of-hospital cardiac arrest cases presumed to be caused by hypothermia.

Epidemiologic factor	Number of cases	Number of all OHCA cases	Proportion in all OHCA, %	Incidence (/100,000 population/year	*P* value for proportion (Fisher exact probability test)
Year					.02
2013	218	125,124	.17%	.17	
2014	235	125,951	.19%	.19	
2015	198	123,421	.16%	.16	
2016	212	123,554	.17%	.17	
2017	239	127,018	.19%	.19	
2018	271	127,718	.21%	.21	
2019	202	126,271	.16%	.16	
Seasons					<.01
Spring	256	215,783	.12%	Undetermined	
Summer	28	172,860	.02%	Undetermined	
Autumn	180	200,898	.09%	Undetermined	
Winter	1111	289,516	.38%	Undetermined	
Weekday					
Weekend	437	256,620	.17%	Undetermined	
Others	1138	622,437	.18%	Undetermined	
Region					<.01
Hokkaido	129	38,832	.33%	2.4	
Tohoku	194	75,668	.26%	2.2	
Kanto	434	290,619	.15%	1.0	
Chubu	342	158,185	.22%	1.6	
Kinki	186	148,042	.13%	.80	
Chugoku	141	49,032	.29%	1.9	
Sikoku	43	28,030	.15%	1.1	
Kyushu	106	90,649	.12%	.70	
Time					<.01
Morning	837	288,504	.29%	Undetermined	
Other time of day	738	590,553	.12%	Undetermined	

OHCA = out of-hospital cardiac arrest.

### 3.2. Overview of prehospital defibrillation

**Figure 1.**
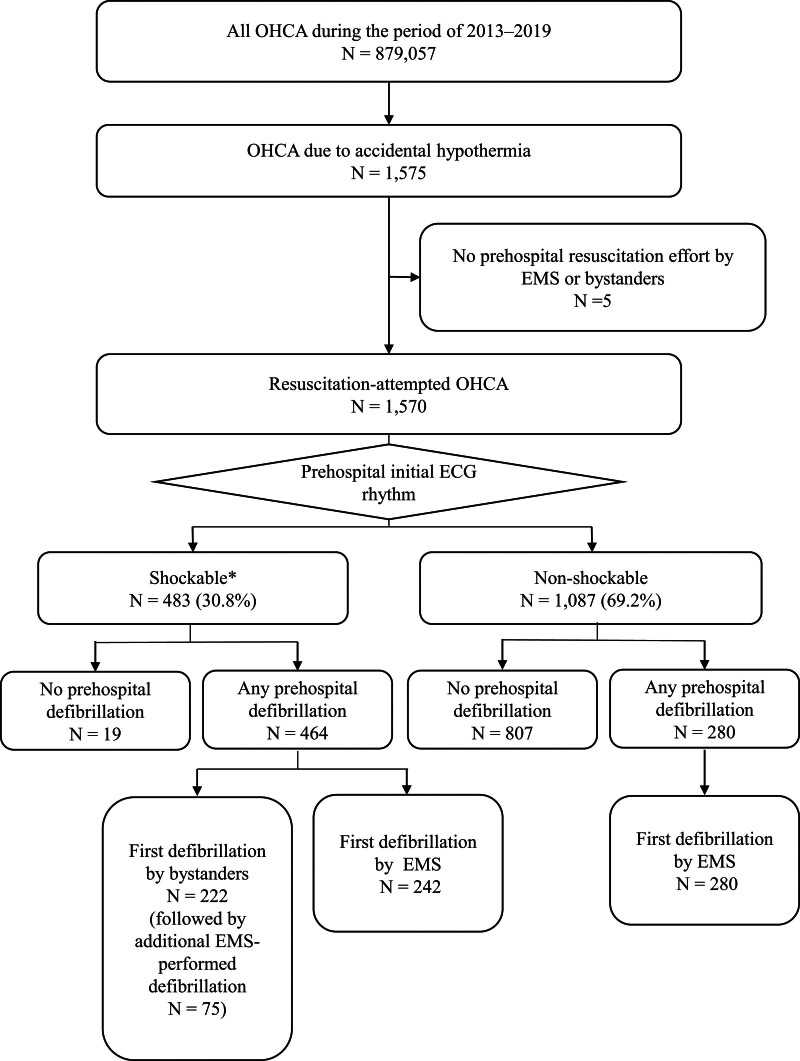
Data selection and overview. * Including cases receiving public access defibrillation before EMS ambulance team arrival. OHCA = out of-hospital cardiac arrest, EMS = emergency medical service.

Of 1570 resuscitation-attempted cases, shockable initial ECG rhythms were recorded in 483 cases (30.8%). prehospital defibrillation was attempted in 464 (96.1%) of 483 cases with shockable rhythms and 280 (25.8%) of 1087 cases with non-shockable initial rhythms. First defibrillation was performed by bystanders, including non-EMS rescue team members, in 222 (47.8%) of 464 cases with shockable rhythms and defibrillation. Approximately 1 third (75/222) of bystander-performed defibrillations were followed up with EMS-performed additional defibrillations (Fig. 1).

After the exclusion of 222 cases that received defibrillation from bystanders using public access defibrillators before the contact of EMS with the patient occurred, the detailed initial ECG rhythms were ventricular fibrillation in 256 cases, ventricular tachycardia in 5 cases, pulseless electric activity in 374 cases, asystole in 471 cases and other non-shockable rhythms (severe bradycardia and non-shockable but ECG recording lacking) in 242 cases.

### 3.3. Characteristics of OHCA among the groups classified based on initial rhythm and prehospital defibrillation

In the groups classified based on initial ECG rhythm, shockable rhythm was associated with higher proportions of prehospital epinephrine administration and advance airway management and a lower incidence of EMS-witnessed OHCA (Table 2). When the initial rhythm was non-shockable, prehospital defibrillation after rhythm conversion was associated with a longer transportation time interval (adjusted OR/10 minutes; 95% CI, 1.10; 1.02–1.19), higher proportions of EMS-witnessed cases (adjusted OR with unwitnessed as reference; 95% CI, 1.92; 1.40–2.64) and prehospital epinephrine administration (95% CI, 1.80; 1.20–2.69). However, epinephrine was rarely administered before the conversion to shockable rhythm (26 out of 161 cases with prehospital epinephrine administration). When the initial rhythm was shockable, some of the characteristics of OHCA differed between the “any defibrillation” and “no defibrillation” groups in univariate analysis, but multivariable logistic regression did not detect any factor associated with prehospital defibrillation (Table 3).

**Table 2:** Comparison of the characteristics of out of-hospital cardiac arrest cases between patients with and without shockable initial electrocardiogram rhythm.

	Prehospital initial ECG rhythm		
Characteristics	Non-shockable (N = 1087)	Shockable (N = 483)	*P* value
Witness status, % (N)			<.01
Unwitnessed	54.6 (593)	57.8 (279)	
Bystander-witnessed	5.1 (55)	10.6 (53)	
EMS-witnessed	40.4 (439)	31.7 (153)	
Age (yr), median (25%–75%)	78 (65–86)	79 (67–86)	.40
Male patient, % (N)	53.6 (583)	52.2 (252)	.59
EMS response time interval (min)*, median (25%–75%)	10 (8–14)	10 (8–14)	.78
Transportation time (min)†, median (25%–75%)	26 (19–36)	26 (19–37)	.63
Prehospital epinephrine administration, % (N)	14.8 (161)	23.6 (114)	<.01
Advanced airway management, % (N)	25.6 (278)	32.9 (159)	<.01
Advanced life support by physician, % (N)	11.2 (122)	13.9 (67)	.14
Physician in ambulance, % (N)	5.6 (61)	5.6 (27)	.99

ECG = electrocardiogram, EMS = emergency medical service.

* Time interval between emergency call and EMS contact to patient.

† Time interval between EMS contact to patient and arrival at hospital.

**Table 3:** Characteristics of OHCA cases classified based on initial rhythm and prehospital defibrillation.

	Prehospital initial ECG rhythm non-shockable			Prehospital initial ECG rhythm shockable		
Characteristics	Any prehospital defibrillation (N = 280)	No prehospital defibrillation (N = 807)	*P* value based on univariate analysis*	Any prehospital defibrillation (N = 464)	No prehospital defibrillation (N = 19)	*P* value based on univariate analysis
Witness status, % (N)			<.01			<.01
Unwitnessed	43.6 (122)	58.4 (471)		59.1 (274)	26.3 (5)	
Bystander-witnessed	3.2 (9)	5.7 (46)		10.8 (50)	5.3 (1)	
EMS-witnessed	53.2 (149)	35.9 (290)		30.2 (140)	68.4 (13)	
Age (yr), median (25%–75%)	79 (66–86)	78 (65–87)	.92	79 (67–86)	84 (71–89)	.16
Male patient, % (N)	50.0 (140)	54.9 (443)	.16	52.2 (242)	52.6 (10)	.97
EMS response time interval (min)†, median (25%–75%)	11 (8–15)	10 (8–14)	.11	10 (8–14)	14 (9–20)	.03
Transportation time (min)‡, median (25%–75%)	30 (22–40)	25 (19–34)	<.01	26 (19–37)	28 (21–39)	.48
Prehospital epinephrine administration, % (N)	23.2 (65)	11.9 (96)	<.01	24.1 (112)	10.5 (2)	.17
Advanced airway management, % (N)	30.0 (84)	24.0 (194)	.05	33.8 (157)	10.5 (2)	.03
Advanced life support provided by a physician, % (N)	11.4 (32)	11.2 (90)	.90	13.2 (61)	31.6 (6)	.02
Physician in ambulance, % (N)	5.0 (14)	5.8 (47)	.61	5.0 (23)	21.1 (4)	<.01

ECG = electrocardiogram, EMS = emergency medical service, OHCA = out of-hospital cardiac arrest.

* In multivariable logistic regression analysis, prehospital defibrillation after rhythm conversion was associated with higher proportions of EMS-witnessed cases (adjusted odds ratio, 1.92; 95% confidence interval [CI], 1.40–2.64), prehospital epinephrine administration (adjusted odds ratio, 1.80; 95% CI, 1.20–2.69) and longer transportation time interval (adjusted odds ratio for each 10 minutes; 1.10; 95% CI, 1.02–1.19), with unwitnessed OHCA as reference.

† Time interval between emergency call and EMS contact with the patient.

‡ Time interval between EMS contact with the patient and arrival at the hospital.

Additional analyses of the comprehensive database from 2015 to 2019 revealed no significant difference in the proportion of outdoor or home OHCA, transportation to high-level emergency hospitals, or active rewarming during transportation either between the 2 major groups with and without shockable initial rhythms or among minor subgroups with and without any prehospital defibrillation (Table S1, Supplemental Digital Content, http://links.lww.com/MD/I851).

### 3.4. Outcomes of OHCA

**Figure 2.**
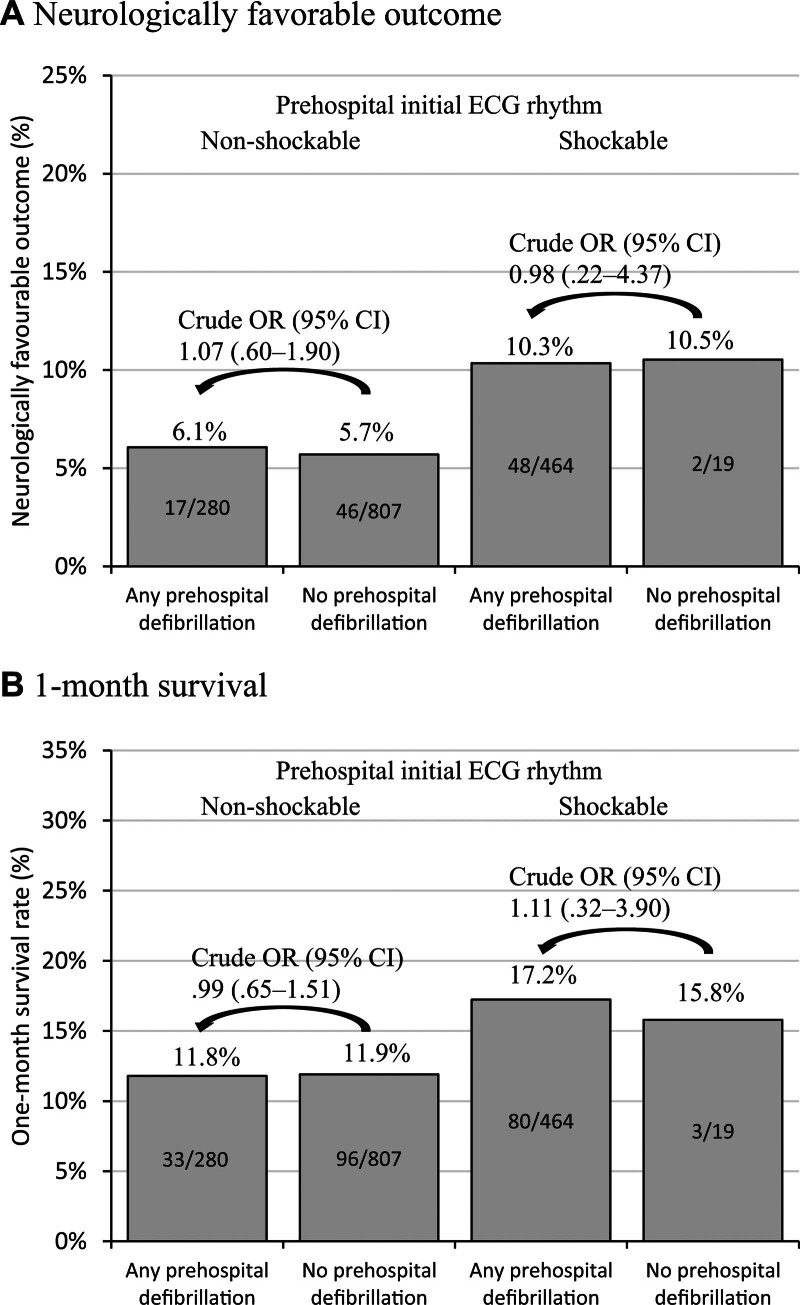
Neurologically favorable outcome and 1-month survival. ECG = electrocardiogram, OR = odds ratio, CI = confidence interval.

When the initial rhythm was non-shockable, the rates of neurologically favorable 1-month survival and 1-month survival were 6.1% and 11.8%, respectively, in 280 cases that received any defibrillation, and 5.7% and 11.9%, respectively, in 807 cases that received no defibrillation. When the initial rhythm was shockable, these rates were 10.3% and 17.2%, respectively, in 464 cases that received any defibrillation, and 10.5% and 15.8%, respectively, in 19 cases that received no defibrillation (Fig. 2). There were no significant differences in outcomes between the “any defibrillation” and “no defibrillation” groups in the subgroups with shockable/non-shockable initial rhythms.

Both simple binominal logit analysis and multivariable logistic regression analysis for all OHCA cases revealed that shockable initial rhythm but not prehospital defibrillation was associated with the outcome, whereas prehospital defibrillation was not associated with outcome, even when multivariable logistic regression was applied (Table 4). Similar results were obtained by additional analyses of the comprehensive database including information on the level of emergency hospitals transported (Table S2, Supplemental Digital Content, http://links.lww.com/MD/I852). Multiple logistic regression analyses on the comprehensive database revealed that transportation to high-level emergency hospitals was associated with better neurologically favorable outcomes (adjusted OR: 2.94, 95% CI: 1.66–5.21), 1-month survival (3.09, 2.04–4.67).

**Table 4:** Odds ratio of prehospital defibrillation and initial electrocardiogram rhythm for outcomes.

Analysis	OR (95% CI)			
	Neurologically favorable outcome		1-M survival	
	Shockable initial ECG rhythms	Prehospital defibrillation	Shockable initial ECG rhythms	Prehospital defibrillation
Simple binominal logit analysis for all OHCAs	1.80 (1.05–3.09)	1.06 (.62–1.81)	1.54 (1.02–2.32)	1.00 (.67–1.49)
Adjusted by multivariable logistic regression analysis for all OHCAs	2.76 (1.47–5.18)	.82 (.44–1.53)	2.15 (1.35–3.45)	.77 (.49–1.21)

Multivariable logistic regression analysis included patient sex, age, witnessed status (unwitnessed, bystander-witnessed, and EMS-witnessed), initial ECG rhythm (shockable or not), prehospital defibrillation, and time intervals of call receipt-to-arrival at patient (EMS response time interval) and arrival at patient to-arrival at hospitals (EMS transportation time interval).

CI = confidence interval, ECG = electrocardiogram, OHCA = out of-hospital cardiac arrest, OR = odds ratio.

### 3.5. Number of defibrillation and outcomes

The rate of neurologically favorable 1-month survival was highest when the number of defibrillations was 2 (Table 5). No survivor was recorded when the number of defibrillations was > 6 in cases with non-shockable initial rhythms. However, statistically, there were no significant associations between the number of defibrillations and the outcomes either for all OHCA cases or in the 2 subgroups (shockable/non-shockable initial rhythm). Furthermore, multivariable logistic regression analysis that included the “no defibrillation” group revealed that any number of defibrillations did not induce better outcomes compared with no defibrillation (Table 6).

**Table 5:** Association of number of defibrillations with outcomes.

Groups and outcomes	Number of defibrillations					*P* based on univariate analysis*
	1	2	3	4–6	>6	
All OHCA cases, N	375	119	93	105	52	
1-month survival, % (N)	15.2% (57)	20.2% (24)	15.1% (14)	11.4% (12)	11.5% (6)	.71
Neurologically favorable 1-month survival, % (N)	7.7% (29)	15.1% (18)	8.6% (8)	6.7% (7)	5.8% (3)	.80
Cases with non-shockable initial rhythms, N	137	46	46	38	13	
1-month survival, % (N)	13.9% (19)	8.7% (4)	10.9% (5)	13.2% (5)	.0% (0)	.90
Neurologically favorable 1-month survival, % (N)	5.1% (7)	8.7% (4)	6.5% (3)	7.9% (3)	.0% (0)	.34
Cases with shockable initial rhythms, N	238	73	47	67	39	
1-month survival, % (N)	16.0% (38)	27.4% (20)	19.2% (9)	10.5% (7)	15.4% (6)	.70
Neurologically favorable 1-month survival, % (N)	9.25 (22)	19.2% (14)	10.6% (5)	6.0% (4)	7.7% (3)	.90

OHCA, out of-hospital cardiac arrest.

* Cochran-Armitage test.

**Table 6:** Adjusted odds ratios for outcomes with no defibrillation as reference.

Groups and outcomes		Adjusted OR (95% CI) for number of defibrillations				
	0 (no defibrillation)	1	2	3	4–6	>6
1-month survival	Reference	.71 (.43–1.17)	1.2 (.65–2.24)	.77 (.36–1.63)	.74 (.34–1.61)	.79 (.29–2.21)
Neurologically favorable 1-month survival	Reference	.64 (.32–1.29)	1.6 (.74–3.46)	.95 (.36–2.49)	.51 (.17–1.55)	.35 (.07–1.75)

CI = confidence interval, OR = odds ratio.

## 4. Discussion

After analyzing the FDMA data, we found that OHCA from exposure and hypothermia occurred everywhere in Japan, with the exception of Okinawa Island. The incidence of hypothermic OHCA was found to be highest in severe winter. Consistent with the findings of a previous investigation,^[17]^ hypothermic OHCA was most frequent in the morning. According to a recent study in urban areas of Japan, hypothermia mostly occurs in the elderly and in outdoor settings and the in-hospital death rate of these patients was 26.3%.^[18]^ Similar findings have been reported in other countries.^[19,20]^

According to the Swiss staging system, the stage of hypothermia is defined based on body temperature, and cardiac arrest with hypothermia is associated with a core temperature of < 24°C.^[21]^ It is likely that most of the hypothermic OHCA cases included in this study had severe hypothermia (with a core temperature of < 24°C), although the core temperature is not measured by EMS personnel in Japan.

In general OHCA, initial cardiac rhythm, especially defibrillation waveforms, is an important factor of OHCA and a good indicator of a favorable 1-month neurologic status.^[22]^ A shockable initial rhythm affects the outcome regardless of the age of the patient.^[23]^ In the present study, hypothermic OHCA had a shockable initial rhythm rate of 30.8% (483/1570). In OHCA due to hypothermia, as with general OHCA, we found that shockable initial rhythm was a good indicator of a neurologically favorable outcome at 1 month after the event. However, defibrillation before arrival at the hospital in the case of a shockable initial rhythm was not associated with a neurologically favorable outcome, regardless of the number of defibrillations. Additional analyses of the comprehensive database revealed similar results regardless of location, level of hospital to which patients were transferred and actively warming during transfer.

In this study, defibrillation was attempted in 280 out of the 1087 cases with non-shockable initial rhythm after rhythm conversion. Furthermore, epinephrine administration was unlikely to be associated with rhythm conversion because epinephrine is rarely administered before rhythm conversion. The administration of epinephrine to patients with hypothermic OHCA varies according to the guidelines, and concerns were raised that epinephrine may accumulate to toxic levels and that drug responsiveness may decrease owing to decreased drug metabolism. Therefore, there is a possibility that the instructing physicians did not indicate epinephrine administration prior to defibrillation. Hypothermia and rewarming from hypothermia are known to induce Ventricular Fibrillation.^[24,25]^ It is likely that rewarming efforts during CPR contribute to the rhythm conversion.

In hypothermic OHCA, unlike general OHCA, defibrillation after rhythm conversion was not associated with better outcomes. Defibrillation is less likely to be successful in patients with severely hypothermic OHCA,^[26]^ and epinephrine and other antiarrhythmic drugs are considered to be less effective below a core body temperature of 30°C,^[27]^ which may further reduce the effect of premorbid defibrillation. In the present study, the core body temperature at the time of defibrillation was not measured, but defibrillation was likely to have been performed in patients with low body temperature. Defibrillation after rhythm conversion might not have been associated with better outcomes as defibrillation is refractory at low body temperatures.

Additional analysis of the comprehensive database revealed that transportation to high-level emergency hospitals was associated with better outcomes. Patients with hypothermia treated with extracorporeal life support are known to have favorable outcomes.^[28]^ Higher-level emergency hospitals are more likely to provide care, including the use of extracorporeal life support, which may have contributed to better outcomes.

Hypothermia can protect the brain and heart by inducing hibernation,^[29,30]^ and effective rewarming and optimization of myocardial perfusion are key to successful defibrillation.^[26,30]^ Numerous studies have reported that prehospital return of spontaneous circulation and body temperature are not associated with survival with neurologically favorable outcomes.^[6,20]^ Currently, it is recommended that defibrillation be performed up to 3 times if the temperature is below 30°C and that the procedure be postponed until rewarming to 30°C or higher thereafter.^[4,30]^ However, the number of repetitions at temperatures at which defibrillation can be performed effectively in severe hypothermia has not yet been established.^[3,4]^ Additionally, because defibrillation causes secondary myocardial damage,^[31]^ repeated ineffective defibrillation is unlikely to lead to good results. Accordingly, in the present study, increasing the number of defibrillations did not improve patient prognosis. The results of our study show that a neurologically favorable prognosis is related only to the shockable initial rhythm detected by the ECG, not defibrillation during transport. Therefore, in hypothermic OHCA, it may be appropriate to refrain from defibrillation before arriving at the hospital or to extend the defibrillation interval, when comprehensive treatment, including more active rewarming of the patient, can be performed. In addition, transport to a high-level acute care hospital may be appropriately considered despite prolonged transport because several reports demonstrated that neurological favorable recovery is possible after hours of cardiopulmonary resuscitation.^[5]^

## 5. Limitations

This study has several limitations. First, Japanese ambulance crews cannot measure core body temperature, thus data on the core body temperature of patients are unavailable. Therefore, severity assessment by core body temperature was impossible. Second, data on comorbidities that affected the outcome were insufficient. Third, the database did not include detailed information on bystander characteristics, such as age, sex, quality of bystander CPR and etiology of cardiac arrest. Fourth, the database did not include details of in-hospital care, including in-hospital medication. Furthermore, treatment and intervention for patients with hypothermia were individually determined by each hospital and physician, allowing the possibility that the decision influenced the prognosis. Fifth, the instructions provided by the physician for procedures performed by EMS personnel during patient transport (epinephrine administration and defibrillation, among others) might have affected the prognosis because they were determined by each instructing physician. Sixth, this study was based on data on whether non-shockable initial rhythms were subsequently defibrillated. Therefore, defibrillation was not performed in all cases with a non-shockable initial rhythm after shockable rhythm conversion. Seventh, the integrity, validity and ascertainment bias of the data are potential limitations, as with all observational studies.

## 6. Conclusion

In hypothermic OHCA, shockable initial rhythm may be associated with better neurologically favorable outcomes, regardless of defibrillation performed prior to arrival at the hospital. In addition, transport to a high-level acute care hospital may be appropriately considered despite prolonged transport. Further investigation, including core temperature data in analyses, is necessary to determine the benefit of prehospital defibrillation in hypothermic OHCA.

## Acknowledgments

Thanks to THE ALL -Japan Utstein Registry of the Fire and Disaster Management Agency for providing the database.

## Author contributions

**Conceptualization:** Tomoyuki Ushimoto, Hideo Inaba.

**Data curation:** Hideo Inaba.

**Formal analysis:** Hideo Inaba.

**Investigation:** Tomoyuki Ushimoto, Kenshi Murasaka, Yukihiro Wato, Hideo Inaba.

**Methodology:** Tomoyuki Ushimoto, Hideo Inaba.

**Project administration:** Tomoyuki Ushimoto, Yukihiro Wato, Hideo Inaba.

**Supervision:** Yukihiro Wato, Hideo Inaba.

**Validation:** Tomoyuki Ushimoto, Hideo Inaba.

**Writing – original draft:** Tomoyuki Ushimoto, Hideo Inaba.

**Writing – review & editing:** Tomoyuki Ushimoto, Kenshi Murasaka, Hideo Inaba.

## Supplementary Material




