# Supplementary material for: Associations between prehospital defibrillation and outcomes of out-of-hospital cardiac arrests presumed to be caused by hypothermia: A nationwide observational study with epidemiological analysis
Source: Medicine (Baltimore). 2023 Apr 28;102(17):e33618. doi: 10.1097/MD.0000000000033618 (PMC10146074; doi:10.1097/MD.0000000000033618)
Supplement: Supplementary file 1 [file medi-102-e33618-s001.pdf]

Supplemental Table 1. Additional characteristics of out-of-hospital cardiac arrest among 4 groups classified by initial rhythm and prehospital defibrillation during the period of 2015-2019

Characteristics	Prehospital initial ECG rhythm non-shockable				Prehospital initial ECG rhythm shockable		
	Any prehospital defibrillation (N = 194)	No prehospital defibrillation (N = 585)	P value by univariate analysis		Any prehospital defibrillation (N = 293)	No prehospital defibrillation (N = 14)	P value by univariate analysis
Location, % (N)							
Outdoor	38.7 (75)	37.4 (219)	.76		41.0 (120)	35.7 (5)	.70
Home	68.0 (132)	65.8 (385)	.57		62.5 (183)	71.4 (10)	.46
Transportation to high-level emergency hospitals [a)], % (N)	49.0 (95)	42.6 (249)	.12		40.6 (119	42.9 (6)	.87
Active rewarming during transportation, % (N)	61.4 (105)	56.0 (280)	.22		52.9 (130)	66.7 (8)	.35

a) Hospitals providing highly advanced emergency care including extracorporeal circulation

ECG, electrocardiogram
