# Supplementary material for: Associations between prehospital defibrillation and outcomes of out-of-hospital cardiac arrests presumed to be caused by hypothermia: A nationwide observational study with epidemiological analysis
Source: Medicine (Baltimore). 2023 Apr 28;102(17):e33618. doi: 10.1097/MD.0000000000033618 (PMC10146074; doi:10.1097/MD.0000000000033618)
Supplement: Supplementary file 2 [file medi-102-e33618-s002.pdf]

Supplemental Table 2. Odds ratio of prehospital defibrillation and initial electrocardiogram rhythm for outcomes

Analysis	OR (95% CI)				
	Neurologically favorable outcome			1-M survival	
	Shockable initial ECG rhythms	Prehospital defibrillation		Shockable initial ECG rhythms	Prehospital defibrillation
Simple binominal logit analysis for all OHCAs	1.91 (1.01–3.61)	1.22 (.65–1.81)		1.41 (0.88–2.26)	1.21(.77–1.90)
Adjusted by multivariable logistic regression analysis for all OHCAs	3.59 (1.61–8.00)	.82 (.37–1.81)		2.24 (1.25–4.02)	0.86 (.49–1.49)

Multivariable logistic regression analysis included the level of hospitals transported, patient sex, age, witnessed status (unwitnessed, bystander-witnessed, and EMS-witnessed), initial ECG rhythm (shockable or not), prehospital defibrillation, and time intervals of call receipt-to-arrival at patient (EMS response time interval) and arrival at patient-to-arrival at hospitals (EMS transportation time interval). Multiple logistic regression analysis on the comprehensive database revealed that transportation to high-level emergency hospitals was associated with better outcomes: adjusted OR (95% CI) for neurologically favorable outcomes and 1-M survival, 2.94 (1.66-5.21) and 3.09 (2.04-4.67), respectively.

OR, odds ratio; CI, confidence interval; OHCA, out-of-hospital cardiac arrest; ECG, electrocardiogram; EMS, emergency medical service
